# A set of empirical equations describing the observed colours of metal–anodic aluminium oxide–Al nanostructures

**DOI:** 10.3762/bjnano.11.64

**Published:** 2020-05-13

**Authors:** Cristina V Manzano, Jakob J Schwiedrzik, Gerhard Bürki, Laszlo Pethö, Johann Michler, Laetitia Philippe

**Affiliations:** 1 Empa, Swiss Federal Laboratories for Materials Science and Technology, Laboratory for Mechanics of Materials and Nanostructures, Feuerwerkerstrasse 39, CH-3602 Thun, Switzerland

**Keywords:** anodic aluminium oxide (AAO) films, anodization, structural colours, reflectance, polar coordinates, plasmonic effects

## Abstract

Structural colours have received a lot of attention regarding the reproduction of the vivid colours found in nature. In this study, metal–anodic aluminium oxide (AAO)–Al nanostructures were deposited using a two-step anodization and sputtering process to produce self-ordered anodic aluminium oxide films and a metal layer (8 nm Cr and 25, 17.5 and 10 nm of Au), respectively. AAO films of different thickness were anodized and the *Yxy* values (*Y* is the luminance value, and *x* and *y* are the chromaticity values) were obtained via reflectance measurements. An empirical model based on the thickness and porosity of the nanostructures was determined, which describes a gamut of colours. The proposed mathematical model can be applied in different fields, such as wavelength absorbers, RGB (red, green, blue) display devices, as well as chemical or optical sensors.

## Introduction

Recently, the reproduction of the vivid colours found in nature has received increasing interest [1]. These colours appear when light interacts with periodic structures. With regard to this, anodic aluminium oxide films play an important role. There are different approaches in obtaining brilliant colours using AAO films, such as the use of new anodization electrolytes (etidronic acid) [2], the use of pulsed anodization [3], and the deposition of a metal layer onto the surface of AAO–Al films [4]. In particular, metal–AAO–Al nanostructures exhibit structural colours that can find applications as wavelength absorbers [5], in RGB display devices [6], and as chemical [7] or optical sensors [8]. It is essential to develop a model that allows for the determination of the colours (RGB or *Yxy* values of CIE 1931 colour space) of these nanostructures based on the morphological parameters of the AAO films.

The colour observed in metal–AAO–Al nanostructures depends on the morphological parameters of the AAO films and on the nature and film thickness of the metal deposited onto the AAO–Al films. In a previous study by our group, the effects of morphological parameters (pore diameters, interpore distance, porosity, and nanostructure order) on the colours and the effective refractive index of AAO films were studied on metal–AAO–Al nanostructures [4]. Thickness and porosity are the two main structural parameters that affect the observed colours and the effective refractive index of the AAO films. By tuning either one of these parameters, or by changing the nature of the coating, it is possible to obtain the all colours of the visible spectrum. Various studies can be found in which the colour is changed by changing the used metal (Pt/Pd, Al, Cr, Ag, or Au) [9–10], by using carbon [5], or by changing the thickness of the metal film [9,11–12].

There are two published studies in which wavelength values were generated by using a model that could predict colours by taking into account the morphological parameters of AAO films and the deposited metal layer [13–14]. One work put forward an equation to determine the thickness of the AAO layer, which exhibits strong absorption of a given wavelength [13]. The other work proposed an empirical equation, also based on wavelengths, that enables the design of colours by choosing a specific thickness [14]. Also, there is a model based on optical measurements (ellipsometry integrating indirectly thickness, porosity) in combination with total reflectance measurements to access the colours, in terms of L*a*b* values, of AAO–Al nanostructures filled with a metal after electrodeposition [15]. Human vision is trichromatic, i.e., the retina contains three types of colour receptor cells, also known as cones: a) S cones, short-wavelength cones or blue cones; b) M cones, middle-wavelength cones or green cones and c) L cones, long-wavelength cones or red cones. A full plot of all visible colours forms a 3D space [16]. The CIE 1931 colour space was created by the International Commission on Illumination (CIE) in 1931 [12] in order to determine a colour model that represents the human colour vision. This model can be described as RGB colour space or as *Yxy* colour space. It is important to bring forward a model that describes a wide range of colours using not only wavelengths, but also tristimulus values. To date, a model to estimate the colours observed in metal–AAO–Al nanostructures, where the metal is deposited on the top of the AAO–Al films, using RGB values or *Yxy* values from reflectance measurements and morphological properties (thickness and porosity) has not been defined.

The purpose of this work is to obtain an empirical model to estimate a gamut of colours knowing only the thickness and porosity of the AAO films. The model was developed using *Yxy* values measuring only the reflectance, thickness and porosity of the films by means of SEM. To achieve this, AAO films of different thickness were anodized and the obtained *Yxy* values were converted into polar coordinates to determine the relationship between the thickness and the colour range described by the *xy* values. Additionally, the *xy* equations are written in terms of effective refractive index and second anodization time, because there is a dependency between the thickness of AAO films and the effective refractive index, as well as the duration of the second anodization process. The model proposed in this study was defined for two different metals, chromium and gold. The work reported in this manuscript provides a mathematical model to estimate the *xy* values in the CIE 1931 colour diagram by only measuring the thickness and porosity of the AAO films.

## Results and Discussion

### AAO film surface morphology

**Figure 1:**
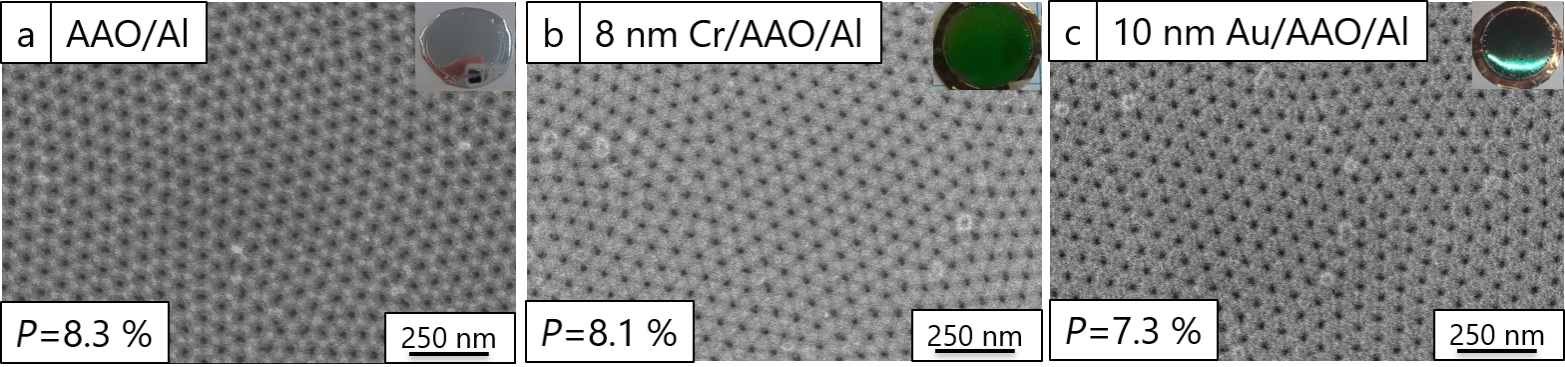
(a) FESEM image of an AAO–Al film, (b) FESEM image of an 8 nm Cr–AAO–Al film, and (c) FESEM image of a 10 nm Au–AAO–Al film. The insets show photos of the nanostructures.

As mentioned above, thickness and porosity are the two morphological parameters of AAO films that have the greatest influence on the colour obtained when depositing a metal layer on top of these nanostructures. Different AAO films were anodized under the same conditions (yielding to the same porosity), changing only the second anodization time (from 120 to 600 s) to obtain different film thicknesses (from 209 ± 12 nm to 380 ± 15 nm). Focused ion beam (FIB) milling and field-emission scanning electron microscopy (FESEM) imaging were used to accurately determine the thickness of the films, similar to a previous study [4]. The porosity, *P*, of the AAO templates is given by the following equation:

[1] $$P=\frac{2\pi }{\sqrt{3}}{\left(\frac{D_{\text{p}}/2}{D_{\mathrm{int}}}\right)}^{2},$$

where *D*_p_ is the pore diameter and *D*_int_ is the interpore distance [17]. The porosity values were found to be 8.3%, 8.1% and 7.3% for AAO–Al films, 8 nm Cr–AAO–Al films and 10 nm Au–AAO–Al films, respectively (Figure 1).

As expected, the porosity of the nanostructures on the surface was smaller after deposition of Cr or Au. The smallest value was achieved after the deposition of 10 nm of Au. It can be concluded that the size of the nanoparticles is bigger for 10 nm Au than for 8 nm Cr. The colour obtained from both metals will vary depending on the thickness of the AAO films, as a result of porosity changes. Figure S1 (Supporting Information File 1) shows FESEM images of AAO–Al films, reflectance spectra before and after the deposition of 25 nm of Au, and the colour diagram of these nanostructures with different porosity volume fractions (9, 26, 31 and 41%). A change in the obtained reflectance and colour is observed for different porosity values. The reflectance spectra (Figure S1e, Supporting Information File 1) show a blueshift with increasing porosity. In the colour diagram (Figure S1f, Supporting Information File 1), the position of the colours moves counter-clockwise as the porosity increases.

### Reflectance, colour diagram and *xy* values of 8 nm Cr–AAO–Al and 10 nm Au–AAO–Al nanostructures

**Figure 2:**
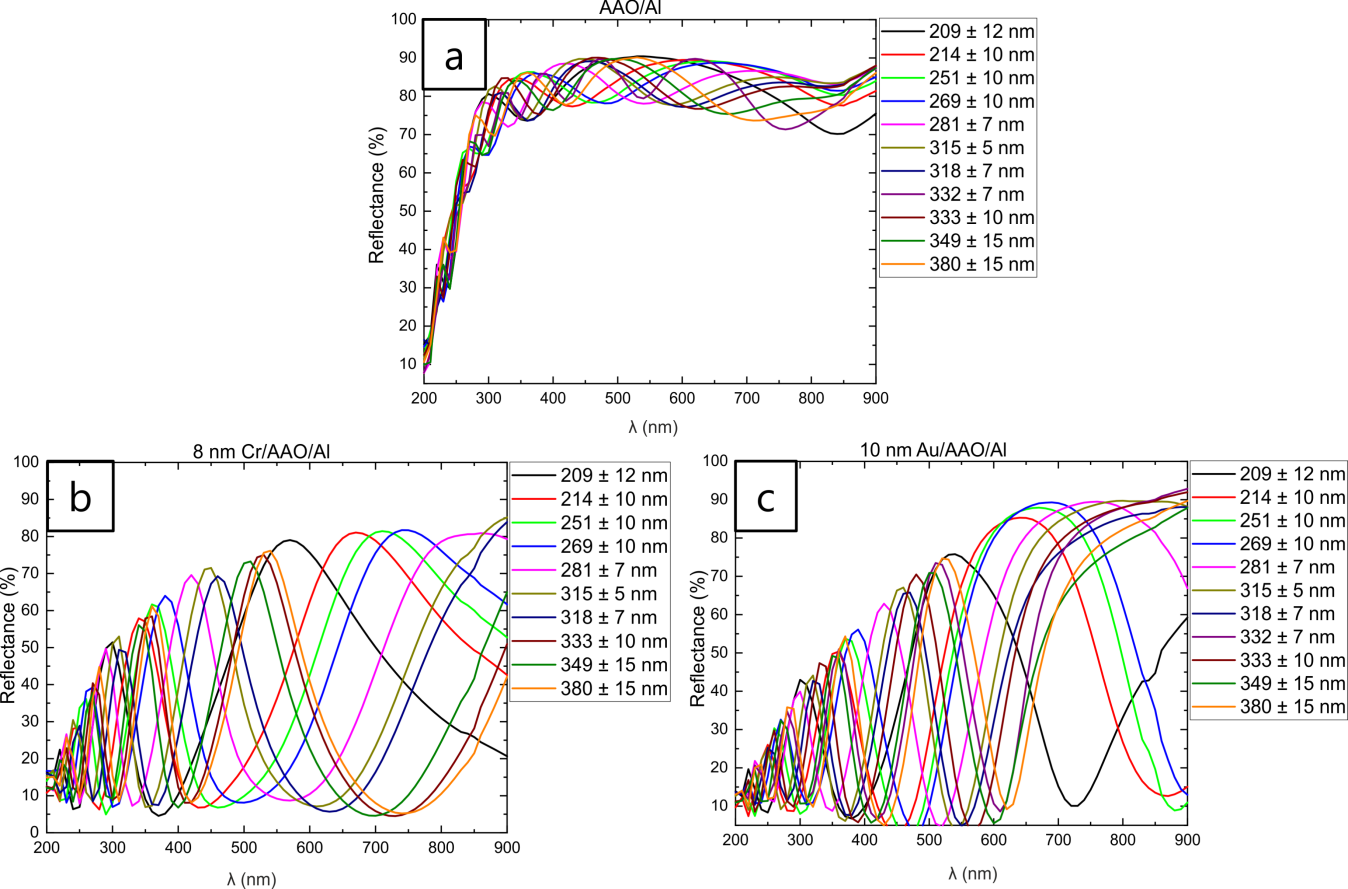
(a) UV–vis reflectance spectra of AAO–Al films, (b) UV–vis reflectance spectra of 8 nm Cr–AAO–Al films, and (c) UV–vis reflectance spectra of 10 nm Au-AAO-Al films with different thickness.

UV–vis reflectance spectra of AAO–Al, 8 nm Cr–AAO–Al films, and 10 nm Au–AAO–Al films with different thickness are shown in Figure 2a, Figure 2b and Figure 2c, respectively.

Figure 2a shows that the number of fringes increases with the increase in thickness. For the same number of fringes, a redshift is observed when the thickness increases. This behaviour has been observed before [18–19]. In both cases, after the deposition of 8 nm of Cr and 10 nm of Au on top of the AAO–Al films (Figure 2b and Figure 2c, respectively), a blueshift and a decrease in the reflectance is observed due to plasmonic effects [4,11,13]. The wavelength of each sample and consequently the colour that the films will exhibit can be obtained from the maximum reflectance. The observed colours, the maximum reflectance wavelengths, and the observed colours from these wavelengths for each thickness value are presented in Table 1.

**Table 1:** AAO thickness, observed colour, wavelength of maximum reflectance, and colour obtained from the wavelength for 8 nm Cr–AAO–Al and 10 nm Au–AAO–Al nanostructures.

**8 nm Cr–AAO–Al nanostructures**			

thickness (nm)	observed colour	λ_max_ (nm)	colour from λ_max_

209 ± 12	yellow	569	yellow
214 ± 10	orange	671	red
251 ± 10	orange	708	red
269 ± 10	pink	744	red
281 ± 7	violet	420	violet
315 ± 5	blue	450	blue
318 ± 7	blue-green	460	blue
333 ± 10	green	530	green
349 ± 15	green	509	green
380 ± 15	green	540	green

**10 nm Au–AAO–Al nanostructures**			

thickness (nm)	observed colour	λ_max_ (nm)	colour from λ_max_

209 ± 12	yellow	536	green
214 ± 10	orange	645	red
251 ± 10	orange	667	red
269 ± 10	orange	681	red
281 ± 7	pink	748	red
315 ± 5	pink	458	blue
318 ± 7	blue	483	blue-green
332 ± 7	green	509	Green
333 ± 10	green	511	green
349 ± 15	green	507	green
380 ± 15	green	523	green

As can be seen in Table 1, the colours obtained from the wavelength and the observed colour are not the same for certain film thicknesses. Therefore, the wavelength should not be the only parameter taken into consideration when designing a model for predicting colours. As was mentioned in the Introduction, human vision is trichromatic. The CIE 1931 colour space describes the RGB colour space or *Yxy* colour space for the entire gamut of visible colours using tristimulus values. For colour prediction it is necessary to obtain either *Yxy* values or RGB values. The *Yxy* values were calculated from the reflectance spectra of the AAO–Al, 8 nm Cr–AAO–Al, and 10, 17.5 and 25 nm Au–AAO–Al nanostructures using the CIE 1931 colour space. The CIE *Yxy* space contains two parts: *Y* is the luminance (brightness), and *x* and *y* are values that define chromaticity. It is possible to determine the colour gamut of human vision by only considering the *x* and *y* values.

**Figure 3:**
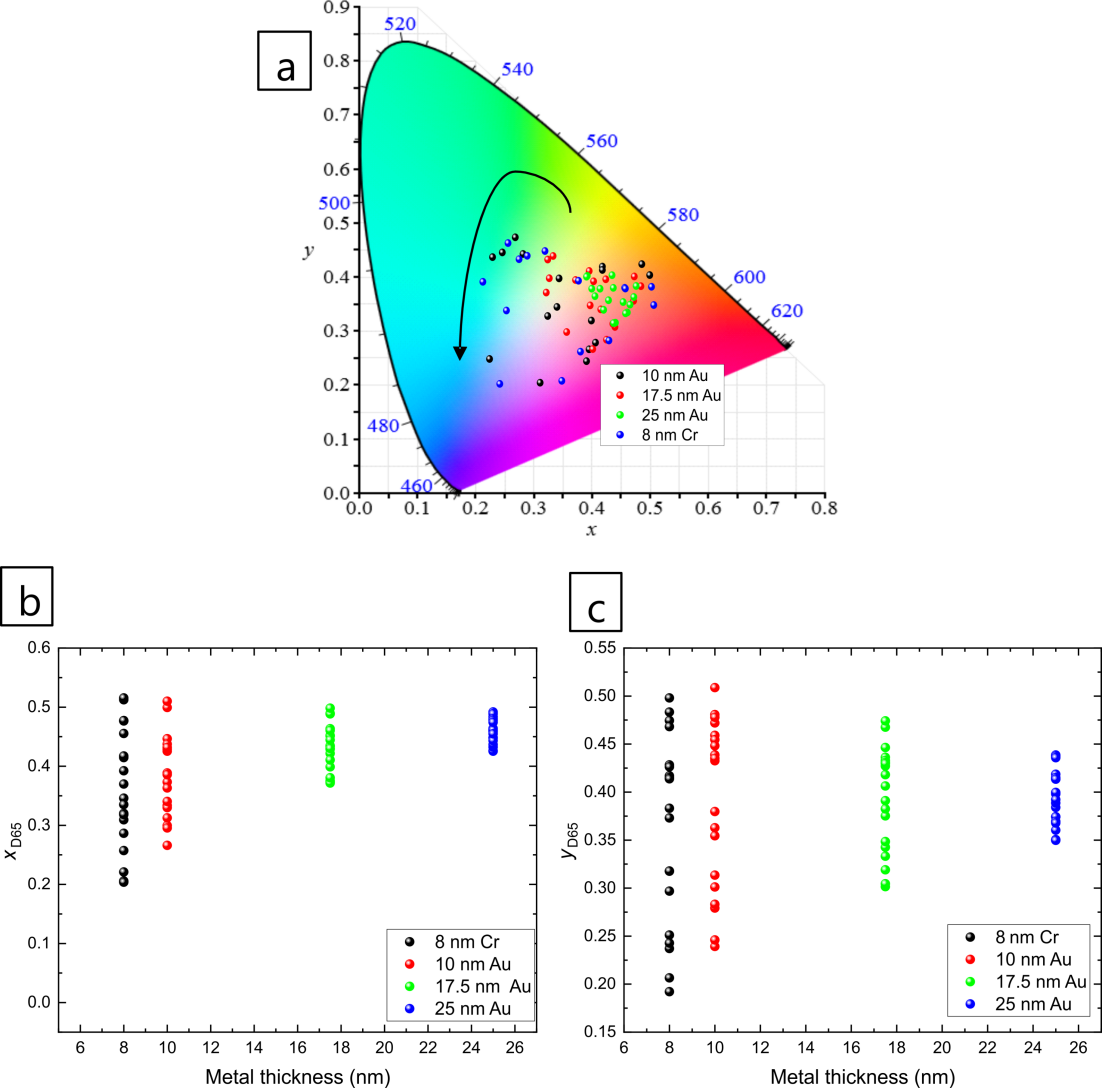
(a) Representation of CIE 1931 colour diagram for different metals (Cr and Au) and different Au thicknesses (10, 17.5 and 25 nm). (b) The values of *x* and (c) the values of *y* as a function of metal thickness. The range of AAO thickness is 209–380 nm for all nanostructures plotted in the figure.

Figure 3a shows the colour coordinates in the CIE 1931 colour diagram for different thicknesses of Cr and Au. Different colours, i.e., *xy* values, were obtained for Cr and Au, as well as for different Au layer thicknesses. It should be noted that in this figure various metal–AAO–Al films (from 209 ± 12 nm to 951 ± 19 nm) are presented in order to show the change of *x* and *y* values when different Au thicknesses are sputtered on AAO–Al films. However, the colour model was designed only with AAO films with a thickness in the range of 209 ± 12 nm to 380 ± 15 nm, as a complete circle in the colour diagram is covered. For thicker samples, more circles are included within the inner part of the colour diagram.

Figure 3 shows that the samples with 17.5 nm Au and 25 nm Au layers do not display a large colour range (*x* and *y* values). For this reason, the colour model was created using the samples with 8 nm Cr or 10 nm Au on top of the AAO–Al nanostructures. The thickness and *x* and *y* values for both samples are presented in Table S1 (Supporting Information File 1).

### Empirical model to obtain the colours observed in Cr/Au–AAO–Al nanostructures

**Figure 4:**
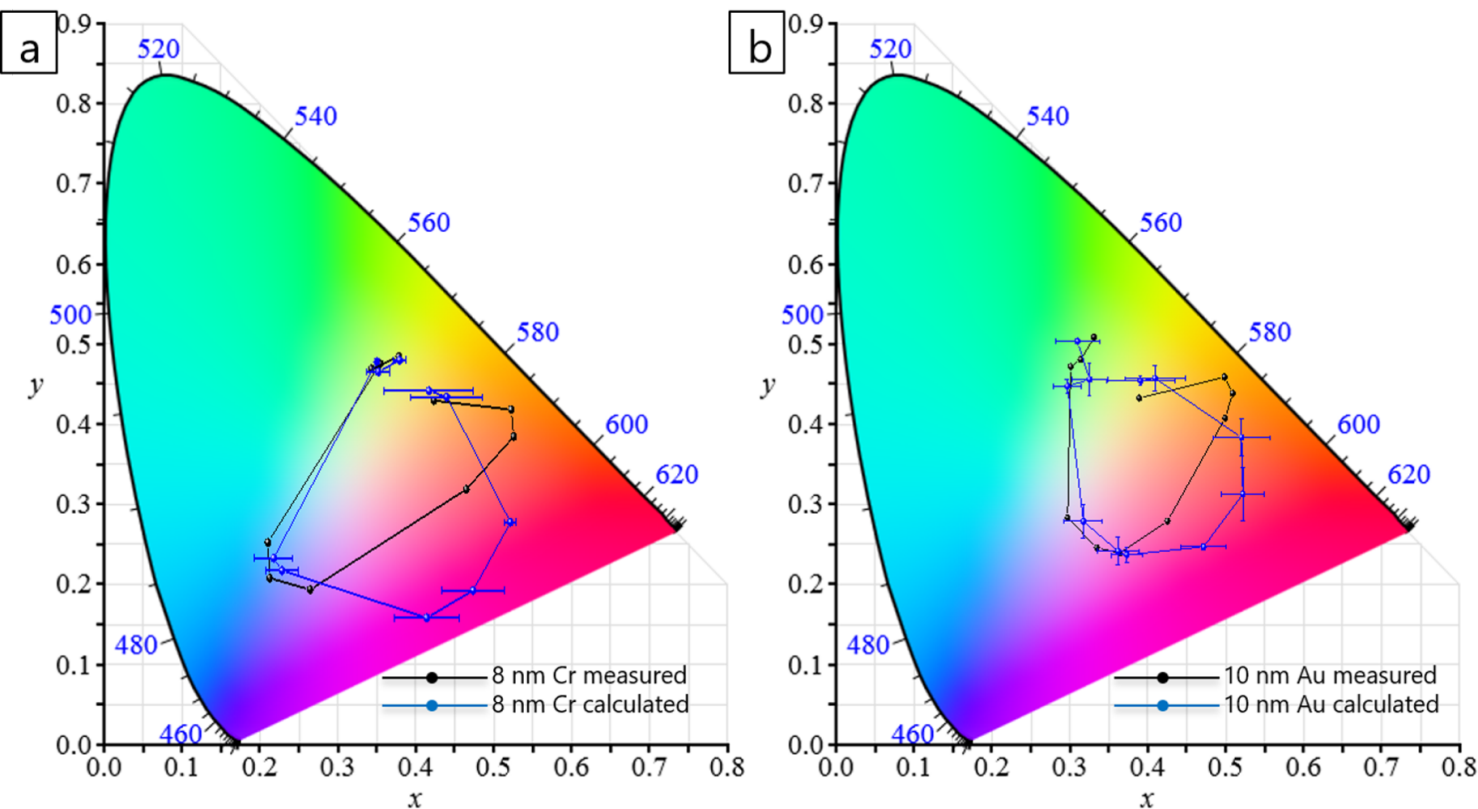
CIE 1931 colour diagrams (a) for 8 nm Cr–AAO–Al and (b) for 10 nm Au–AAO–Al films. Measured values are plotted in black and calculated values in blue.

An empirical model was defined by using the thickness values measured from SEM images and the *x* and *y* values obtained from the reflectance measurements. The proposed model defines a spiral shape. First, Cartesian coordinates (*x, y*) are transformed to polar coordinates (*R*, θ) by applying Equation S1 and Equation S2 (Supporting Information File 1). The values were divided into two different ranges, as the *x* and the *y* coordinates of the nanostructures lie very far from each other in the colour diagram for thicknesses of 209–332 nm and of 332–380 nm, even when the AAO films have very similar thickness (Figure 4). The equations obtained for 8 nm Cr–AAO–Al nanostructures are:

[2]
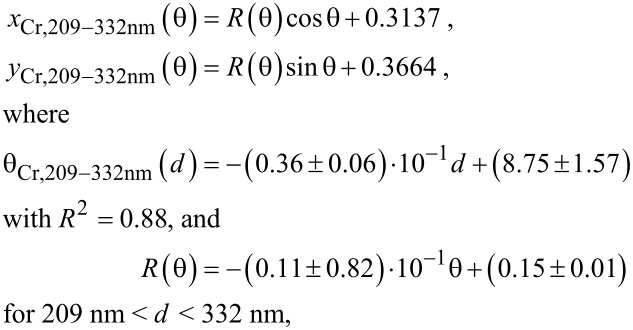


and

[3]
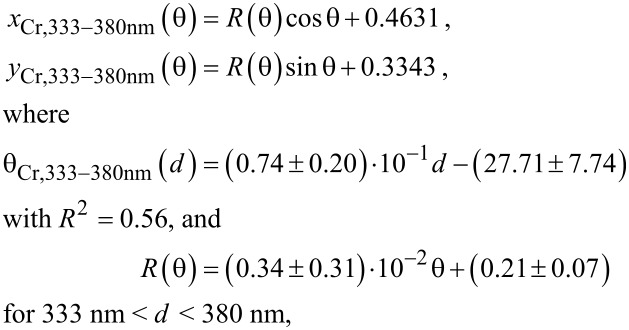


where *d* is the thickness of the AAO film.

The equations obtained for 10 nm Au–AAO–Al nanostructures are given in Supporting Information File 1 (Equation S3 and Equation S4). Note that these equations are valid when AAO–Al films have a porosity of 8.3%. Equations 2 and 3 as well as Equations S3 and S4 are very similar, as the top pore diameter after the deposition of 8 nm Cr (8.1%) and of 10 nm Au (7.3%) is similar and the colours (*x* and *y* values) obtained after the metal deposition process are comparable (see Figure 4 and Table S1, Supporting Information File 1).

In a previous study carried out by our group, the influence of different anodization electrolytes on the effective refractive index and the thickness of AAO films was examined [4]. In this work, more films and thinner films were studied, therefore it is necessary to recalculate the equation for the thickness between 209 ± 12 nm and 951 ± 19 nm. An exponential equation was obtained (see Figure S2 in Supporting Information File 1):

[4] $$n_{\text{eff}}=\left(4.12\pm 1.07\right)e^{\left(\frac{d}{183.84\pm 52.50}\right)}+\left(1.64\pm 0.19\right).$$

The dependency of the effective refractive index on the thickness obtained in the previous study was linear, as the thickness of the AAO films varied between 400 and 1300 nm [4]. Moreover, the thickness can be formulated as a function of the second anodization time, *t*^2nd^, as follows:

[5] $$d=\left(54.23\pm 1.43\right)\cdot t^{\text{2nd}}+\left(90.03\pm 9.31\right).$$

The relationship between the thickness of AAO films and the second anodization time is linear (see Figure S3, Supporting Information File 1). The equations for 8 nm Cr–AAO–Al and for 10 nm Au–AAO–Al nanostructures can now be written as a function of the effective refractive index and second anodization time. These equations are given in Supporting Information File 1, Equations S5–S8.

Figure 4a and Figure 4b show the CIE 1931 colour diagrams obtained from the reflectance measurements and calculated using the equations of the model proposed for 8 nm Cr–AAO–Al and 10 nm Au–AAO–Al nanostructures, respectively. The errors of *x* and *y* values were calculated using the propagation of uncertainties.

The thickness, the *x* and *y* values obtained from reflectance measurements, and the *x* and *y* values calculated using the proposed model are presented in Table S1 (Supporting Information File 1). As can be seen in Figure 4 and Table S1 (Supporting Information File 1), the observed colours are equal to the colours predicted using the model. A certain error is obtained due to the large errors in the thickness measurements of the thin films. The *x* and *y* error values depend strongly on the thickness error as the *x* and *y* values depend on θ and on *R*(θ). θ depends on the film thickness, as shown in Equations 2 and 3 as well as Equations S3 and S4. The model yields a better fit for the samples with 10 nm Au deposited onto the AAO–Al films than for the samples with 8 nm Cr sputtered onto these films. It should be noted that measuring the thickness of the thin films as well as obtaining accurate values is very difficult due to the roughness and large surface area (2.5 cm^2^ in diameter) of the AAO films. This can be seen in the FESEM images of the cross section of an AAO film after FIB cutting (Figure S4, Supporting Information File 1).

**Figure 5:**
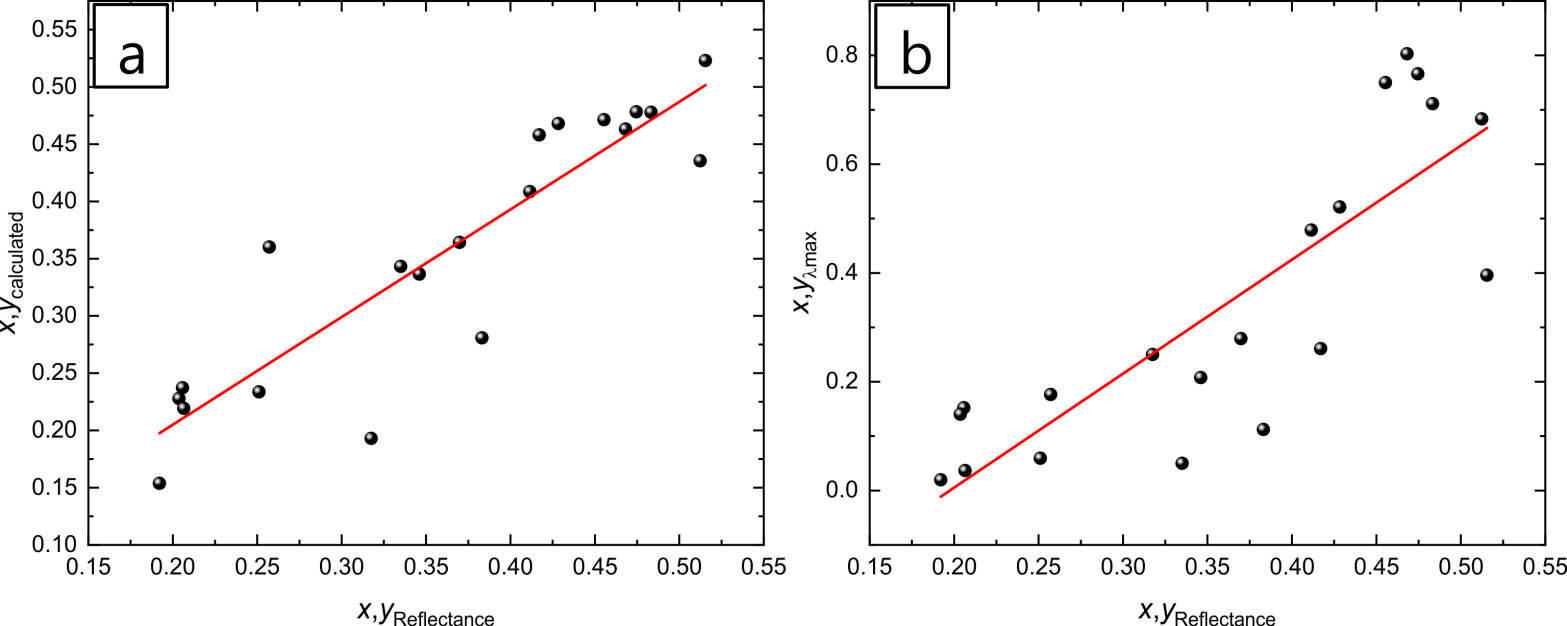
(a) *x* and *y* values calculated from the proposed model as a function of *x* and *y* values obtained from the reflectance measurements. (b) *x* and *y* values calculated from the wavelength of the maximum reflectance as a function of *x* and *y* values obtained from the reflectance measurements.

The *x* and *y* values calculated from the proposed model were plotted as a function of the *x* and *y* values from the reflectance measurements, in order to check the validity of the proposed model (Figure 5a). In order to compare the proposed model in this study and the method using the wavelength of the maximum reflectance, the *x* and *y* values were calculated from the wavelength of the maximum reflectance and were plotted as a function of the *x* and *y* values obtained from the reflectance measurements (Figure 5b).

Values of slope and *R*^2^ of 0.94 ± 0.09 and 0.82, respectively, were obtained by plotting the calculated *x* and *y* values as a function of the *x* and *y* values obtained from the reflectance measurements. When the *x* and *y* values obtained from the wavelength of the maximum reflectance were plotted as a function of the *x* and *y* values from the reflectance measurements, the values of slope and *R*^2^ were 2.09 ± 0.31 and 0.47, respectively. These values corroborate that the mathematical model proposed in this study is more accurate that other equations found in literature, which use wavelengths and not tristimulus values describing a gamut of colours using the CIE 1931 colour space. This empirical model can be applied to any type of material and thickness of metal films deposited on the surface of AAO–Al films. Also, it can be extended to thicker AAO films considering more colours in the inner part of the colour diagram circle.

## Conclusion

8 nm Cr–AAO–Al and 10/17.5/25 nm Au–AAO–Al nanostructures were fabricated by combining sputtering deposition and a two-step anodization process. The AAO films with different thicknesses (from 209 ± 12 nm to 380 ± 15 nm) were anodized and the CIE *Yxy* values were obtained via reflectance measurements. A mathematic model that describes a gamut of colours was proposed using only the thickness and porosity of the AAO films. The model was defined for two different metals, i.e., Cr and Au, finding similar equations for both, as the porosity after the deposition of both metals onto the AAO-Al films was very similar. The proposed model can be used for porous templates with different morphological properties and metal layers deposited onto these templates. This study provides a simple mathematical model that can be useful in different applications, such as wavelength absorbers, RGB display devices, as well as chemical or optical sensors.

## Experimental

### Fabrication of the AAO films

Highly ordered anodic aluminium oxide (AAO) films were fabricated using a two-step anodization process [20–22] under the same conditions that were reported in previous manuscripts of our group [4,18]. The anodization conditions used in this study were as follows: 0.3 M oxalic acid as electrolyte, an applied potential of 40 V and a temperature of 3 °C. Different thicknesses were obtained by applying different second anodization times. Different AAO films were anodized under the same conditions (same porosity and thickness) and the porosity of the films was changed by applying different chemical etching times (from 2 to 8 min) using a 5 wt % H_3_PO_4_ solution at 35 °C. In order to obtain different colours of the nanostructures, an 8 nm thin film of chromium was deposited on top of the AAO films using an Alliance-Concept DP 650 DC magnetron sputtering equipment. 25, 17.5 and 10 nm thin films of gold were deposited on top of the AAO films using a Leica EM ACE600 sputtering equipment. The Au layers were deposited using a sputtering pressure and intensity of 5 × 10^−2^ mbar and 30 mA, respectively, and the Cr layer was deposited using a sputtering pressure and power of 5 × 10^−3^ mbar and 350 W, respectively. The metal–anodic aluminium oxide (AAO)–Al nanostructures were formed on mirror Al, which was obtained via electropolishing an Al foil before the anodization process. Two different metals (Cr and Au) were used in order to obtain different colours of the metal–AAO–Al nanostructures due the different plasmonic effects in both metals. In addition, three different metal thicknesses were studied (25, 17.5 and 10 nm of Au) to show the influence of metal thickness on the observed colour. It is important to note that the AAO–Al nanostructures are exactly the same for the study. In order to obtain nanostructures with different Au thickness, a Au layer was deposited and subsequently removed using an aqueous etching solution of I_2_ and KI before the deposition of the next Au layer with different thickness. After that, the Cr layer was deposited.

### Characterization of AAO films

Morphological characterisation was performed using a field-emission scanning electron microscope (FESEM, Hitachi S-4800) with a 1.5 kV accelerating voltage. The thickness was obtained from sample cross sections fabricated using a focused ion beam (FIB) instrument (TESCAN Lyra, Brno, Czech Republic) with a gallium source at 30 kV and 180–400 pA. 2 µm of platinum was deposited to protect the surface prior to FIB cutting. FESEM images were taken in three different areas of the films and three different measurements were carried out. The thickness was calculated by averaging the nine measurements. Reflectance spectroscopy measurements of the AAO films were carried out using a PerkinElmer Lambda 900 UV–vis spectrophotometer, ranging from 200 to 900 nm using the diffuse mode with integrating sphere. CIE *Yxy* values, which represent a colour model including luminance (*Y*) and chromaticity (*xy)* using the CIE 1931 colour space [12], were obtained from the UV–vis reflectance measurements of the nanostructures according to the detailed description in [23]. For these calculations, only the wavelength range of 400–700 nm was considered.

## Supporting Information

File 1 Additional experimental data.

## References

[1] Kolle M, Salgard-Cunha P M, Scherer M R J, Huang F, Vukusic P, Mahajan S, Baumberg J J, Steiner U (2010). Nat Nanotechnol.

[2] Kikuchi T, Nishinaga O, Natsui S, Suzuki R O (2015). Electrochim Acta.

[3] Ruiz-Clavijo A, Tsurimaki Y, Caballero-Calero O, Ni G, Chen G, Boriskina S V, Martín-González M (2018). ACS Photonics.

[4] Manzano C V, Ramos D, Pethö L, Bürki G, Michler J, Philippe L (2018). J Phys Chem C.

[5] Oller D, Fernandes G E, Siontas S, Xu J, Pacifici D (2016). Mater Res Bull.

[6] Lo P-H, Luo G-L, Fang W (2015). Implementation of nanoporous anodic aluminum oxide layer with different porosities for interferometric RGB color pixels as handheld display application. 28th IEEE International Conference on Micro Electro Mechanical Systems (MEMS).

[7] Guo D-L, Fan L-X, Wang F-H, Huang S-Y, Zou X-W (2008). J Phys Chem C.

[8] Bae K, Lee J, Kang G, Yoo D-S, Lee C-W, Kim K (2015). RSC Adv.

[9] Pashchanka M, Yadav S, Cottre T, Schneider J J (2014). Nanoscale.

[10] Wang X, Zhang H, Zhang D, Ma Y, Fecht H-J, Jiang J Z (2012). Microsc Res Tech.

[11] Xue J, Zhou Z-K, Wei Z, Su R, Lai J, Li J, Li C, Zhang T, Wang X-H (2015). Nat Commun.

[12] Smith T, Guild J (1931). Trans Opt Soc, London.

[13] Choi D, Shin C K, Yoon D, Chung D S, Jin Y W, Lee L P (2014). Nano Lett.

[14] Wang X, Zhang D, Zhang H, Ma Y, Jiang J Z (2011). Nanotechnology.

[15] De Graeve I, Laha P, Goossens V, Furneaux R, Verwimp D, Stijns E, Terryn H (2011). Surf Coat Technol.

[16] Purves D, Augustine G J, Fitzpatrick D (2001). Cones and Color Vision. Neuroscience.

[17] Nielsch K, Choi J, Schwirn K, Wehrspohn R B, Gösele U (2002). Nano Lett.

[18] Manzano C V, Best J P, Schwiedrzik J J, Cantarero A, Michler J, Philippe L (2016). J Mater Chem C.

[19] Xu Q, Sun H-Y, Yang Y-H, Liu L-H, Li Z-Y (2011). Appl Surf Sci.

[20] Masuda H, Fukuda K (1995). Science.

[21] Martín J, Manzano C V, Caballero-Calero O, Martín-González M (2013). ACS Appl Mater Interfaces.

[22] Manzano C V, Martín J, Martín-González M S (2014). Microporous Mesoporous Mater.

[23] Giusti M M, Wrolstad R E, Smith D E, Nielsen S S (2010). Calculation of CIE Color Specifications from Reflectance or Transmittance Spectra. Food Analysis Laboratory Manual.

