# Supplementary material for: A set of empirical equations describing the observed colours of metal–anodic aluminium oxide–Al nanostructures
Source: Beilstein J Nanotechnol. 2020 May 13;11:798–806. doi: 10.3762/bjnano.11.64 (PMC7237813; doi:10.3762/bjnano.11.64)
Supplement: File 1 — Additional experimental data. [file Beilstein_J_Nanotechnol-11-798-s001.pdf]

BEILSTEIN JOURNAL OF NANOTECHNOLOGY

Supporting Information

for

A set of empirical equations describing the observed colours of metal–anodic aluminium oxide–Al nanostructures

Cristina V. Manzano, Jakob J. Schwiedrzik, Gerhard Bürki, Laszlo Pethö, Johann Michler and Laetitia Philippe

Beilstein J. Nanotechnol. **2020,** *11,* 798–806. doi:10.3762/bjnano.11.64

Additional experimental data

License and Terms: This is a supporting information file under the terms of the Creative Commons Attribution License (http://creativecommons.org/licenses/by/4.0). Please note that the reuse, redistribution and reproduction in particular requires that the authors and source are credited.
The license is subject to the *Beilstein Journal of Nanotechnology* terms and conditions: (https://www.beilstein-journals.org/bjnano)

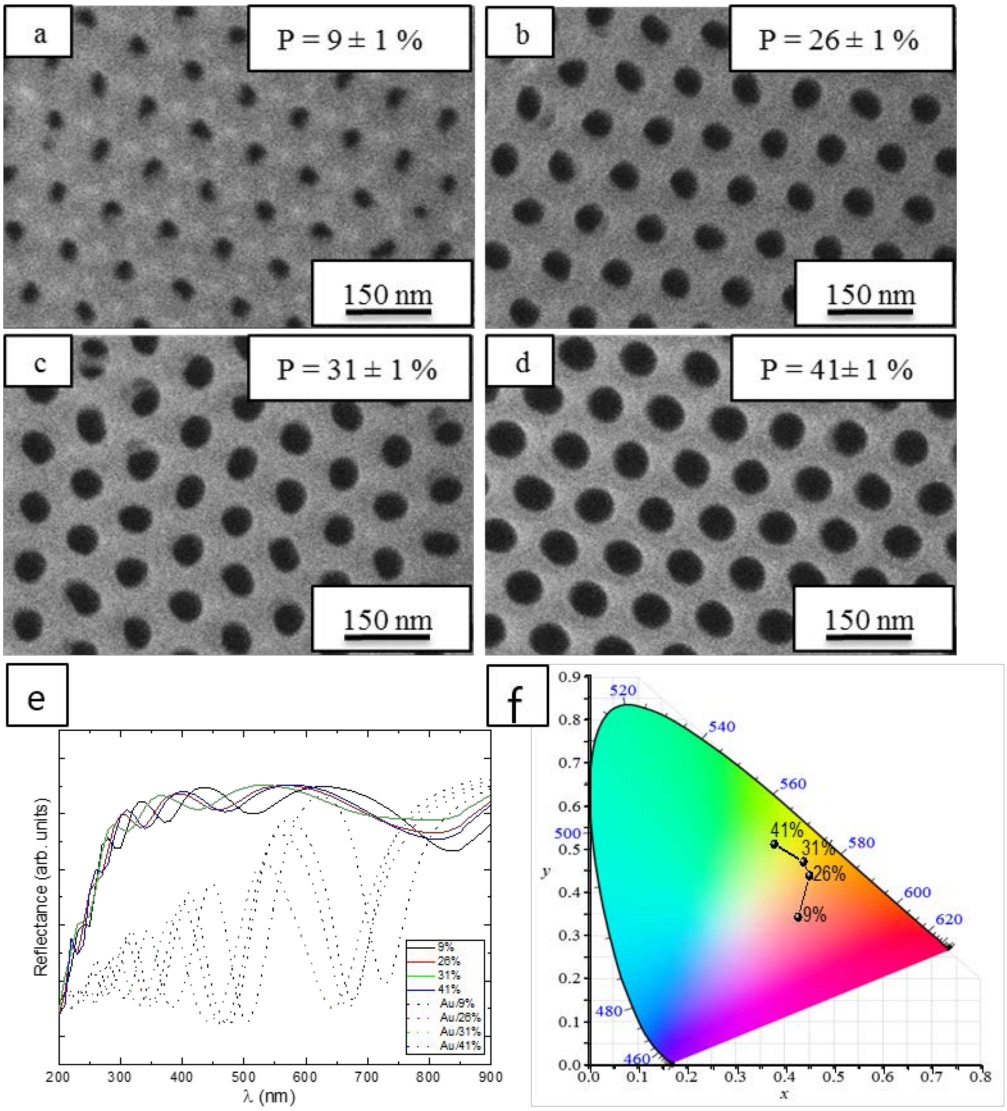

Figure S1: FE-SEM micrographs of AAO films anodized in 0.3 M oxalic acid under 40 V at 3 °C with different porosity after different periods of chemical etching time: (a) 2 min, (b) 4 min, (c) 6 min, and (d) 8 min. (e) UV–vis reflectance spectra of AAO–Al films with different porosity before (continuous lines) and after (dotted lines) deposition of 25 nm Au. (f) Colour diagram of 25 nm Au–AAO–Al films with different porosity.

Equations for transforming Cartesian coordinates (x,y) to polar coordinates (R, θ):

$$R = \sqrt{(x - x_m)^2 + (y - y_m)^2}$$

(S1)

$$\theta = arctan\left(\frac{y - y_m}{x - x_m}\right)$$

where $arctan$ is:

$$\text{arctan (y,x)=}\left(\begin{array}{c} arctan\left(\frac{y}{x}\right)\ if\ x > 0 \\ arctan\left(\frac{y}{x}\right) + \pi\ if\ x < 0\ and\ y \geq 0 \\ arctan\left(\frac{y}{x}\right) - \pi\ if\ x < 0\ and\ y < 0 \\ \frac{\pi}{2} if\ x = 0\ and\ y > 0 \\ -\frac{\pi}{2} if\ x = 0\ and\ y < 0 \\ undefined\ if\ x = 0\ and\ y = 0 \end{array}\right)$$

(S2)

The equations obtained for 10 nm Au–AAO–Al nanostructures are:

$x_{\text{Au,209-332nm}}$ (θ) = R(θ)·cos θ + 0.3483,

$y_{\text{Au,209-332nm}}$ (θ)= R(θ)·sin θ + 0.4145, (S3)

where $\theta_{\text{Au,209-332nm}}(d) = -(0.35 \pm 0.04)\cdot 10^{-1}\cdot d + (9.14 \pm 1.20)$ with $R^2 = 0.92$

and $R(\theta) = -(0.27 \pm 0.58)\cdot 10^{-2}\cdot\theta + (0.11 \pm 0 .01)$ for 209 nm < d < 332 nm,

and

$x_{\text{Au,333-380nm}}$ = R(θ)·cos θ + 0.3140,

$y_{\text{ Au,333-380nm}}$ = R(θ)·sin θ + 0.4872, (S4)

where $\theta_{\text{Au,333-380nm}}(d) = (0.66 \pm 0.25)\cdot 10^{-1}\cdot d - (23.76 \pm 8.80)$ with $R^2 = 0.77$

and $R(\theta) = (9.31 \pm 0.90)\cdot 10^{-2}\cdot\theta + (0.29 \pm 0.17)\cdot 10^{-1}$ for 333 nm < d < 380 nm.

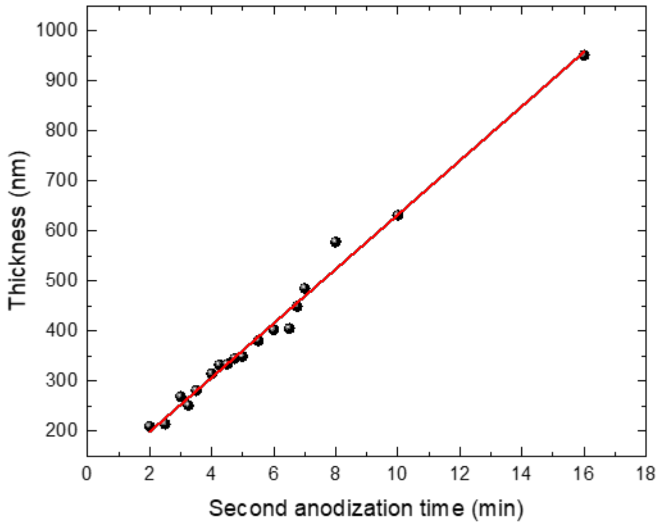

Figure S2: Thickness as a function of the second anodization time for AAO films anodized in an aqueous solution of 0.3M oxalic acid under 40 V at 3 °C.

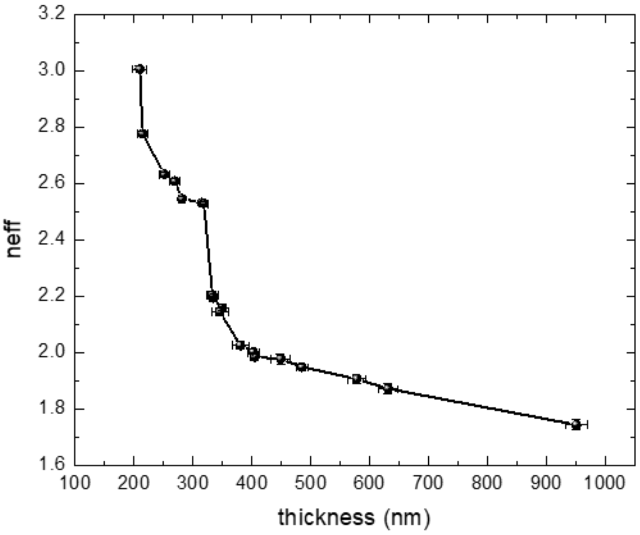

Figure S3: Effective refractive index as a function of the thickness of AAO films anodized in an aqueous solution of 0.3M oxalic acid under 40 V at 3 °C.

The equations for 8 nm Cr–AAO–Al nanostructures can now be written as a function of the effective refractive index and the second anodization time. The value of θ as a function of the effective refractive index (n_{eff}) is:

$$\theta_{Cr,209\text{-}332nm}(n_{\mathrm{eff}}) = -0.36\cdot10^{-1}\cdot\left[(-183.84 \pm 52.50)\cdot ln\left[\frac{neff-(1.64\pm0.19)}{4.12\pm1.07}\right]\right] + 8.75$$

for 209 nm < d < 332 nm, and (S5)

$$\theta_{Cr333\text{-}380nm}(n_{\mathrm{eff}}) = -0.74\cdot10^{-1}\cdot\left[(-183.84 \pm 52.50)\cdot ln\left[\frac{neff-(1.64\pm0.19)}{4.12\pm1.07}\right]\right] + 27.71$$

for 333 nm < d < 380 nm.

The value of θ as a function of the second anodization time, t^{2nd}, is:

$$\theta_{Cr,209\text{-}332nm}(t^{2nd}) = -0.36\cdot10^{-1}\cdot[(54.23 \pm 1.43)\cdot t^{2nd} + (90.03 \pm 9.31)] + 8.75$$

for 209 nm < d < 332 nm, and (S6)

$$\theta_{Cr,333\text{-}380nm}(t^{2nd}) = -0.74\cdot10^{-1}\cdot[(54.23 \pm 1.43)\cdot t^{2nd} + (90.03 \pm 9.31)] + 27.71$$

for 333 nm < d < 380 nm.

The equations for 10 nm Au–AAO–Al nanostructures can now be written as a function of the effective refractive index and the second anodization time. The value of θ as a function of the effective refractive index (n_{eff}) is:

$$\theta_{Au,209\text{-}332nm}(n_{eff}) = -0.35\cdot10^{-1}\cdot\left[(-183.84 \pm 52.50)\cdot ln\left[\frac{neff-(1.64\pm0.19)}{4.12\pm1.07}\right]\right] + 9.14$$

for 209 nm < d < 332 nm, and (S7)

$$\theta_{Au,333\text{-}380nm}(n_{eff}) = 0.66\cdot10^{-1}\cdot\left[(-183.84 \pm 52.50)\cdot ln\left[\frac{neff-(1.64\pm0.19)}{4.12\pm1.07}\right]\right] - 23.76$$

for 333 nm < d < 380 nm.

The value of θ as a function of the second anodization time, t^{2nd}, is:

$$\theta_{Au,209\text{-}332nm}(t^{2nd}) = -0.35\cdot10^{-1}\cdot[(54.23 \pm 1.43)\cdot t^{2nd} + (90.03 \pm 9.31)] + 9.14$$

for 209 nm < d < 332 nm, and (S8)

$$\theta_{Au,333\text{-}380nm}(t^{2nd}) = 0.66\cdot10^{-1}\cdot[(54.23 \pm 1.43)\cdot t^{2nd} + (90.03 \pm 9.31)] - 23.76$$

for 333 nm < d < 380 nm.

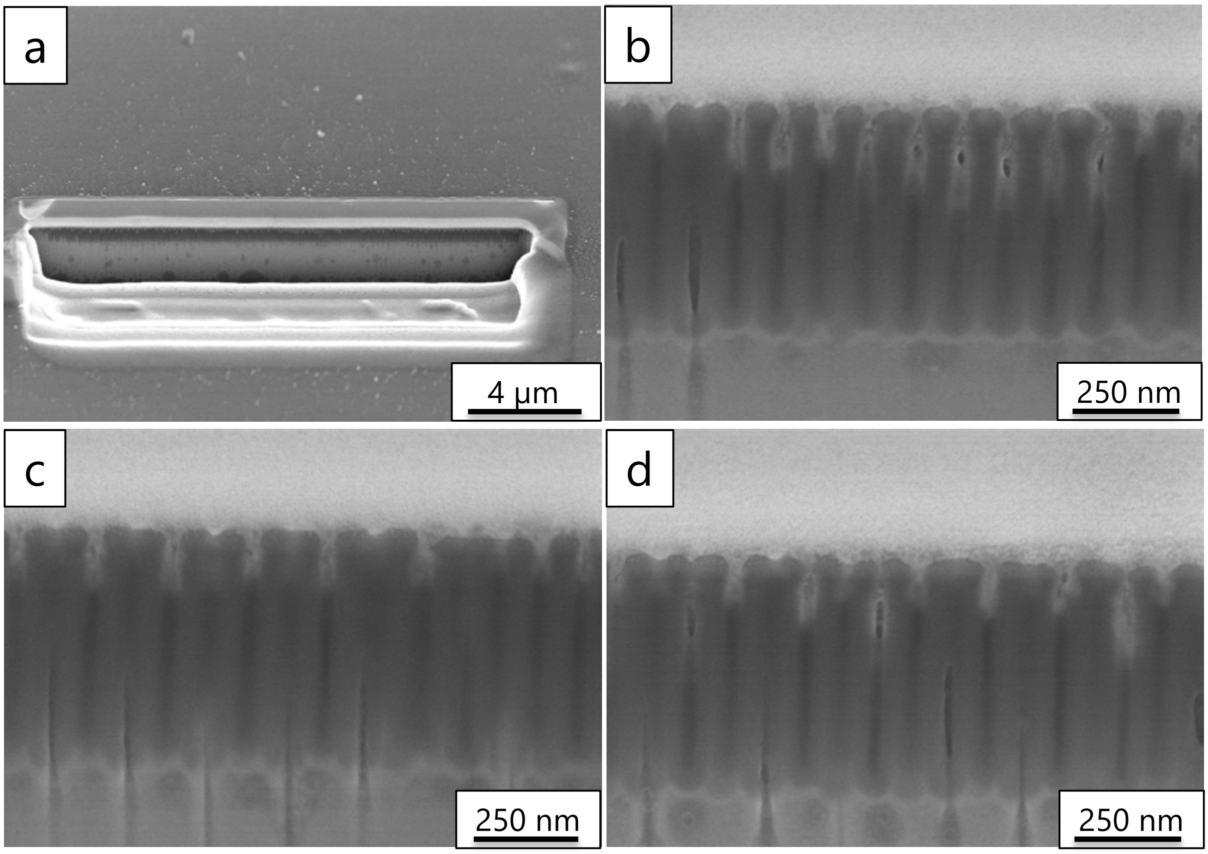

Figure S4: FE-SEM micrographs of AAO films: (a) cross section of an AAO film after FIB cutting, (b–d) cross-sectional images of an AAO film after FIB cutting showing different parts of the sample.

Table S1: AAO thickness, *x* and *y* values obtained from the wavelength of the maximum reflectance, *x* and *y* values calculated from the reflectance measurements and *x* and *y* values calculated from the proposed model for 8 nm Cr–AAO–Al and 10 nm Au–AAO–Al nanostructures.

**8 nm Cr–AAO–Al nanostructures**							
thickness (nm)	λ_{max} (nm)	$x_{\lambda max}$	$y_{\lambda max}$	$x_{Reflectance}$	$y_{Reflectance}$	x_{model}	y_{model}
209 ± 12	569	0.4788	0.5212	0.4115	0.4283	0.4086	0.4680
214 ± 10	671	0.6833	0.2608	0.5124	0.4170	0.4355	0.4580
251 ± 10	708	0.3958	0.1122	0.5155	0.3831	0.5230	0.2808
269 ± 10	700	0.7500	0.2500	0.4554	0.3175	0.4714	0.1930
281 ± 7	420	0.1765	0.0197	0.2572	0.1921	0.3601	0.1538
315 ± 5	450	0.1523	0.0367	0.2058	0.2065	0.2373	0.2192
318 ± 7	460	0.1402	0.0594	0.2037	0.2511	0.2279	0.2336
333 ± 10	530	0.2076	0.7661	0.3460	0.4745	0.3365	0.4782
349 ± 15	509	0.0499	0.8029	0.3350	0.4682	0.3432	0.4631
380 ± 15	540	0.2793	0.7111	0.3698	0.4833	0.3642	0.4778
**10 nm Au–AAO–Al nanostructures**							
thickness (nm)	λ_{max} (nm)	$x_{\lambda max}$	$y_{\lambda max}$	$x_{Reflectance}$	$y_{Reflectance}$	x_{model}	y_{model}
209 ± 12	536	0.2789	0.7115	0.3886	0.4326	0.3877	0.4653
214 ± 10	645	0.7056	0.2944	0.4994	0.4590	0.4086	0.4682
251 ± 10	667	0.6381	0.2399	0.5100	0.4387	0.5284	0.3860
269 ± 10	681	0.7059	0.2941	0.5003	0.4078	0.5287	0.3113
281 ± 7	748	0.7500	0.2500	0.4254	0.2792	0.4741	0.2441
315 ± 5	458	0.1392	0.0591	0.3633	0.2392	0.3723	0.2360
318 ± 7	483	0.1149	0.1092	0.3334	0.2460	0.3607	0.2410
332 ± 7	509	0.1392	0.0591	0.2955	0.2932	0.3164	0.2792
333 ± 10	511	0.0723	0.2289	0.2995	0.4720	0.2870	0.4645
349 ± 15	507	0.0056	0.6751	0.3126	0.4808	0.3048	0.4564
380 ± 15	523	0.1259	0.8094	0.3299	0.5088	0.3145	0.4873
